# Investigation of the Protective Effect of *Centella asiatica* Against Doxorubicin-Induced Testicular Damage in Rats

**DOI:** 10.3390/ijms27177883

**Published:** 2026-09-03

**Authors:** Fevzi Bedir, Zeynep Suleyman, Huseyin Kocaturk, Mehmet Sefa Altay, Ferda Keskin Cimen, Durdu Altuner, Mansura Babayeva, Halis Suleyman

**Affiliations:** 1 Department of Urology, Health Sciences University, Erzurum Regional Training and Research Hospital, Erzurum 25240, Türkiye; kocaturk78@hotmail.com (H.K.); memsefaaltay@gmail.com (M.S.A.); 2 Department of Internal Medicine Nursing, Faculty of Health Sciences, Erzincan Binali Yildirim University, Erzincan 24030, Türkiye; zeynep1105@gmail.com; 3 Department of Pathology, Faculty of Medicine, Erzincan Binali Yildirim University, Erzincan 24030, Türkiye; ferdakeskin@hotmail.com; 4 Department of Pharmacology, Faculty of Medicine, Erzincan Binali Yildirim University, Erzincan 24030, Türkiye; durdualtuner@gmail.com (D.A.); halis.suleyman@gmail.com (H.S.); 5 Department of Internal Medicine, Azerbaijan Medical University, Baku AZ1022, Azerbaijan; dr.babayevamansura@gmail.com

**Keywords:** doxorubicin, *Centella asiatica*, rat, testicular, reproduction

## Abstract

Doxorubicin (DOX) is an antineoplastic agent commonly used in cancer therapy that is known for its testicular toxicity. DOX-induced testicular injury involves inflammation, reactive oxygen species, and oxidative stress. *Centella asiatica* exhibits anti-inflammatory and antioxidant properties. The present experimental study aimed to determine the potential protective efficacy of *Centella asiatica* in a rat model of doxorubicin-induced testicular toxicity. Rats were randomly assigned to four groups: healthy control (HC), CA, DOX, and CA + DOX (CADX). An oral dose of 200 mg/kg *Centella asiatica* was administered to the CA and CADX groups. The HC and DOX groups received saline. One hour later, DOX (7.5 mg/kg) was intraperitoneally injected into the DOX and CADX groups on days 1, 4, and 7. *Centella asiatica* markedly attenuated the DOX-induced increase in malondialdehyde, nitric oxide, and pro-inflammatory cytokine levels, while preventing the decrease in superoxide dismutase activity in testicular tissue (*p* < 0.001). *Centella asiatica* reduced the severity of DOX-induced morphological damage in testicular tissue (*p* < 0.05). Moreover, it partially restored reproductive hormone function. In conclusion, *Centella asiatica* demonstrates significant potential in mitigating DOX-induced oxidative damage, attenuating testicular injury and preserving the functional integrity of the male reproductive system.

## 1. Introduction

Doxorubicin (DOX) is an anthracycline antibiotic with broad antineoplastic activity, widely used in the treatment of numerous solid tumors and hematological malignancies [1]. However, its clinical use is limited by its hematological, pulmonary, hepatic, cardiac, renal, and testicular toxicities [2]. DOX may impair spermatogenesis and alter testicular function, eventually leading to infertility [3]. The mechanisms underlying DOX-induced testicular injury involve inflammation, reactive oxygen and nitrogen species (RONS) generation, autophagy, and oxidative stress [4,5]. Oxidative stress occurs when the production of reactive oxygen species exceeds the effectiveness of antioxidant mechanisms. As the testis is a tissue with high metabolic activity and rapid cell turnover, it is particularly vulnerable to oxidative stress; therefore, its intrinsic antioxidant capacity is of critical importance [6]. Oxidative stress and inflammation are among the key determinants of male reproductive disorders. These processes damage intracellular components and compromise reproductive function. Testicular injury can be evaluated using oxidative stress markers, including malondialdehyde (MDA), antioxidant enzyme activity, cytokines (TNF-α, IL-1β, IL-6), and nitric oxide (NO) [7]. The reduction in elevated NO levels has been considered an indicator of improvement in diabetes-induced testicular injury [8]. DOX has been reported to induce oxidative stress in the testes by increasing lipid peroxidation (LPO) and reducing the activity of antioxidant enzymes, including SOD. [9]. Additionally, DOX may impair fertility by disrupting steroidogenic enzymes and altering the balance of hormones associated with the hypothalamic–pituitary–gonadal axis [10]. Antioxidant therapy has been shown to reduce oxidative stress, support spermatogenesis, and improve male fertility [11]. Several studies have demonstrated that antioxidant supplementation attenuates DOX-induced oxidative stress, improves sperm function, and reduces histopathological damage in the testes [12,13,14,15].

*Centella asiatica* is a medicinal plant known for its potent anti-inflammatory and antioxidant effects. It contains various bioactive compounds, including ascorbic acid, asiatic acid, oleanolic acid, stigmasterol, and α-humulene [16]. In vivo and in vitro studies demonstrate that the mitoprotective and antioxidant effects of *Centella asiatica* contribute significantly to its anti-inflammatory and neuroprotective properties [17]. *Centella asiatica* has been reported to exert hepatoprotective effects against cisplatin-induced hepatotoxicity in rats. [18]. The hepatoprotective effect of *Centella asiatica* may be associated with the suppression of Bax and caspase-3 expression and the upregulation of Bcl-2 expression. Furthermore, *Centella asiatica* has been shown to alleviate oxidative stress in a rat model of nephrotoxicity by enhancing antioxidant defenses and reducing lipid peroxidation [19]. In another study, *Centella asiatica* was reported to exert protective effects against D-galactose-induced cognitive impairment, oxidative stress, and neurodegenerative changes in the adult rat brain [20]. Moreover, *Centella asiatica* was also demonstrated to be effective in attenuating pulmonary fibrosis developing in the lungs of bleomycin-treated rats [21]. Triterpenes derived from *Centella asiatica* have been reported to exert potent antioxidant effects in various cell types [22]. Furthermore, asiatic acid, a major active constituent of *Centella asiatica*, has been shown to mitigate DOX-induced oxidative damage through antioxidant pathways. [23]. Based on these findings, the rationale for investigating *Centella asiatica* in DOX-induced testicular toxicity is that oxidative stress, inflammation, and mitochondrial dysfunction, which are key mechanisms underlying DOX-mediated tissue injury, are also recognized targets of *Centella asiatica* activity. Taken together, the literature suggests that *Centella asiatica*, with its strong antioxidant properties, may provide protection against DOX-induced testicular injury. Although the protective effects of *Centella asiatica* have been reported in different tissue injury models, its role in doxorubicin-induced testicular toxicity has not been fully elucidated. This study highlights the protective potential of *Centella asiatica* by evaluating oxidative stress, inflammatory markers, reproductive hormones, and histopathological changes in a rat model. Therefore, the aim of the present study is to investigate the protective effects of *Centella asiatica* against DOX-induced testicular toxicity in a rat model using biochemical and histopathological analyses.

## 2. Results

### 2.1. MDA and SOD Levels in Testicular Tissue

**Figure 1:**
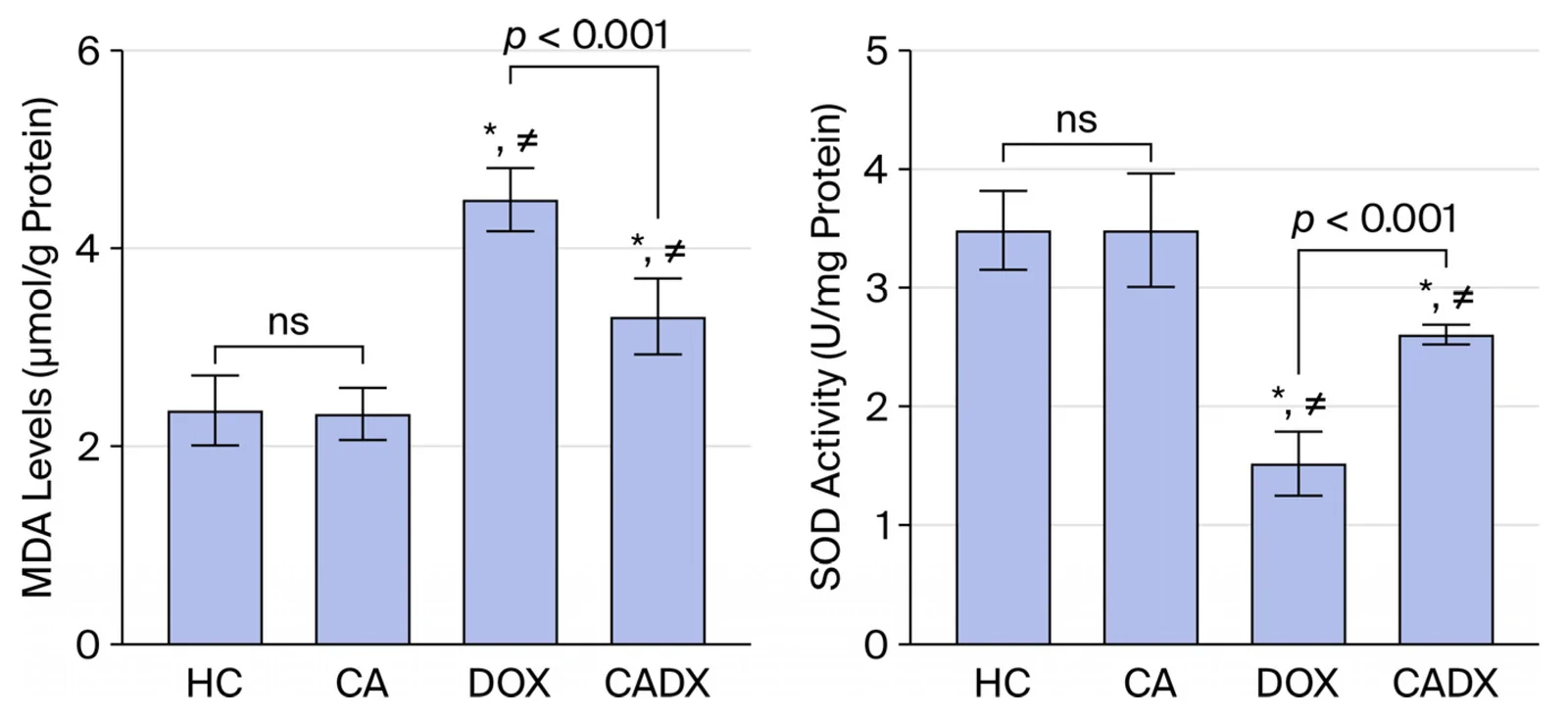
Oxidant and Antioxidant levels in rat testicular tissue. Malondialdehyde (MDA), Superoxide Dismutase (SOD), Healthy Control (HC), *Centella asiatica* (CA), Doxorubicin (DOX), *Centella asiatica* + Doxorubicin (CADX). The bars indicate mean ± standard deviation. *p* < 0.001 * vs. HG, *p* < 0.001 ≠ vs. CA, ns: non-significant.

One-way ANOVA revealed a significant intergroup difference in MDA levels (F(3, 20) = 57.026, *p* < 0.001) ((η^2^ = 0.895), 95% confidence interval (0.749–0.927), the reliability of the observed differences). As depicted in Figure 1, no significant alterations in MDA or SOD levels were observed following *Centella asiatica* treatment alone in comparison with the HC (*p* = 0.997, *p* = 0.995, respectively). However, DOX administration led to a significant increase in MDA levels (*p* < 0.001) alongside a significant reduction in SOD activity (*p* < 0.001). Pre- or co-treatment with *Centella asiatica* significantly prevented these DOX-induced changes, suppressing MDA elevation and restoring SOD activity (*p* < 0.001). One-way ANOVA revealed significant differences in SOD activities across the experimental groups (F(3, 20) = 49.609, *p* < 0.001). The effect size was large (η^2^ = 0.882), while the 95% confidence interval (0.718–0.980) supported the robustness of these findings. Pairwise comparison data, including means and standard deviations, are presented in Table 1.

### 2.2. NO Levels in Testicular Tissue

**Figure 2:**
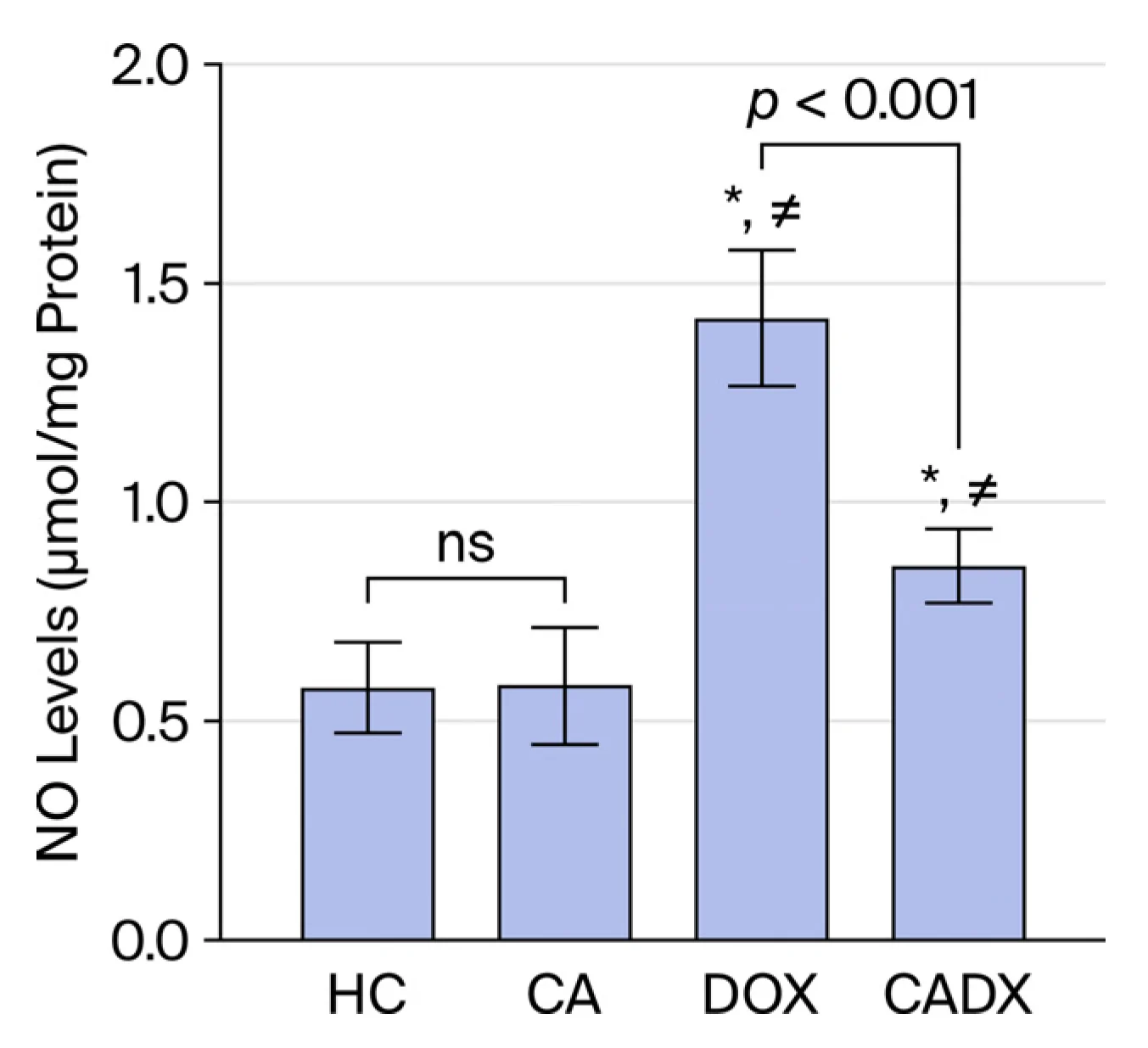
Nitric oxide (NO) levels in rat testicular tissue. Healthy Control (HC), *Centella asiatica* (CA), Doxorubicin (DOX), *Centella asiatica* + Doxorubicin (CADX). The bars indicate mean ± standard deviation. *p* < 0.001 * vs. HG, *p* < 0.001 ≠ vs. CA, ns: non-significant.

Analysis of NO levels indicated a statistically significant variation among the groups (one-way ANOVA, F(3, 20) = 62.878, *p* < 0.001). This difference corresponded to a large effect size (η^2^ = 0.904), with a 95% confidence interval ranging from 0.769 to 0.917. As shown in Figure 2, *Centella asiatica* administration alone did not significantly alter testicular NO levels compared to the HC group (*p* > 0.05). In contrast, doxorubicin monotherapy resulted in a significant elevation of NO levels in testicular tissue relative to the HC group (*p* < 0.001; Table 1). Notably, co-treatment with *Centella asiatica* significantly suppressed this doxorubicin-induced increase in NO levels (*p* < 0.001).

### 2.3. TNF-α, IL-1β, and IL-6 Levels in the Testicular Tissue

**Figure 3:**
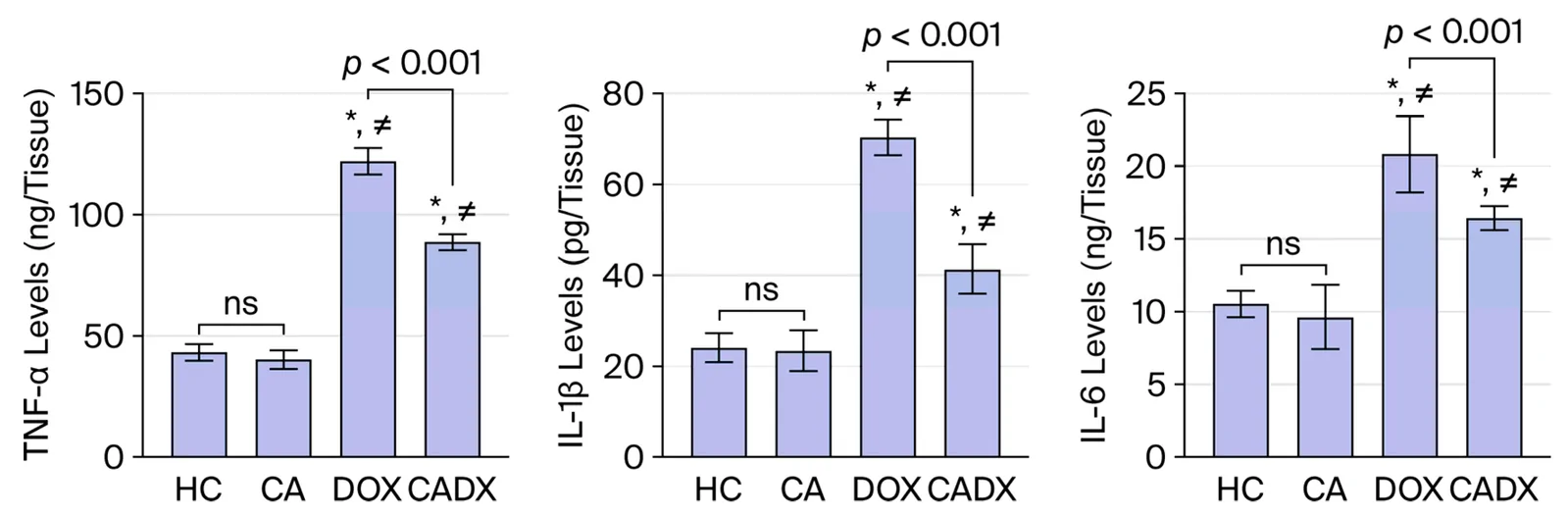
Pro-inflammatory levels in rat testicular tissue. Tumor necrosis factor-alpha (TNF-ɑ), Interleukin-1 beta (IL-1β), Interleukin-6 (IL-6), Healthy Control (HC), *Centella asiatica* (CA), Doxorubicin (DOX), *Centella asiatica* + Doxorubicin (CADX). The bars indicate mean ± standard deviation. *p* < 0.001 * vs. HG, *p* < 0.001 ≠ vs. CA, ns: non-significant.

The comparison of TNF-α levels by one-way ANOVA showed a highly significant group effect (F(3, 20) = 561.489, *p* < 0.001). The effect size was remarkably large (η^2^ = 0.988), and the associated 95% confidence interval (0.971–0.992) reflected a strong and consistent effect. For IL-1β, one-way ANOVA identified significant differences between the groups (F(3, 20) = 154.658, *p* < 0.001). The observed effect was large (η^2^ = 0.959), and the 95% confidence interval (0.898–0.971) further confirmed the stability of the results. Group-related differences in IL-6 levels reached statistical significance according to one-way ANOVA (F(3, 20) = 50.154, *p* < 0.001). A large effect size was obtained (η^2^ = 0.883), and the corresponding 95% confidence interval (0.720–0.918) indicated a reliable group effect. As presented in Figure 3, *Centella asiatica* administration alone did not significantly alter pro-inflammatory cytokine levels compared to the healthy control group (*p* > 0.05). Conversely, doxorubicin monotherapy led to a significant elevation in testicular TNF-α, IL-1β, and IL-6 levels relative to the healthy group (*p* < 0.001). Notably, co-treatment with *Centella asiatica* significantly attenuated this doxorubicin-induced increase in all evaluated cytokines (*p* < 0.001). Comprehensive pairwise comparisons along with the mean ± standard deviation values are detailed in Table 1.

### 2.4. Serum Levels of Reproductive Hormones

**Figure 4:**
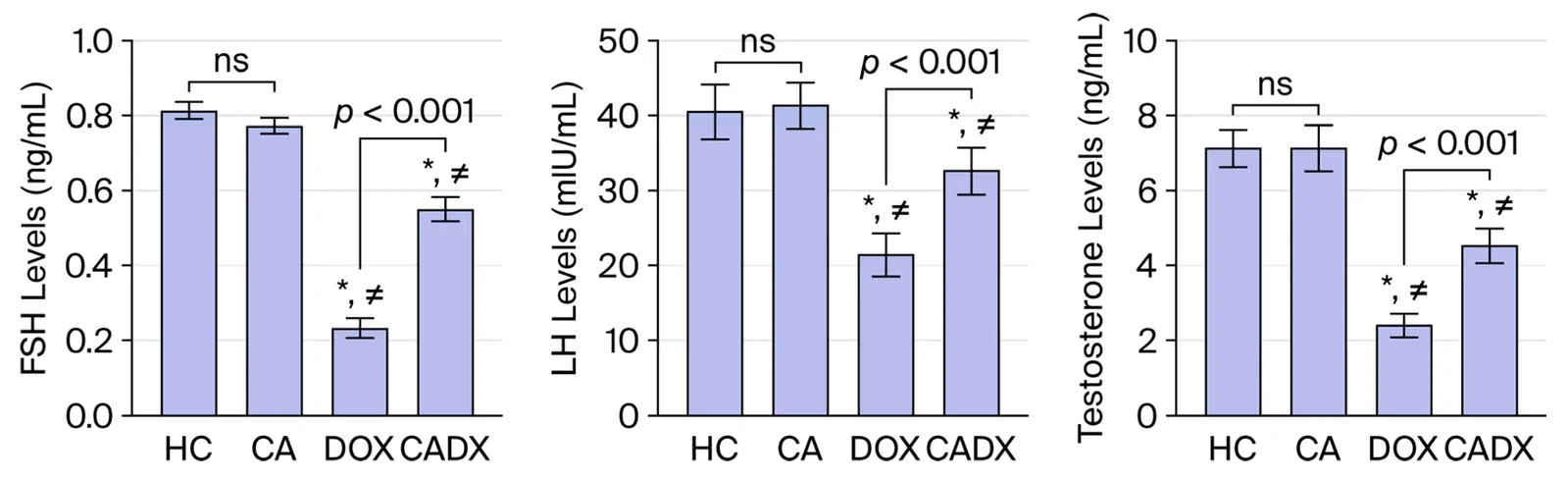
Hormone levels in rat serum. Follicle-stimulating hormone (FSH), Luteinizing hormone (LH), Healthy Control (HC), *Centella asiatica* (CA), Doxorubicin (DOX), *Centella asiatica* + Doxorubicin (CADX). The bars indicate mean ± standard deviation. *p* < 0.001 * vs. HG, *p* < 0.001 ≠ vs. CA, ns: non-significant.

FSH concentrations differed significantly among the experimental groups (one-way ANOVA, F(3, 20) = 587.606, *p* < 0.001). The calculated effect size was large (η^2^ = 0.989), and the 95% confidence interval (0.972–0.992) demonstrated the consistency of the observed differences. A one-way ANOVA performed for LH levels demonstrated significant differences across the groups (F(3, 20) = 50.504, *p* < 0.001). The effect size was large (η^2^ = 0.883), with a 95% confidence interval of 0.722–0.919, indicating a robust statistical effect. The second analysis of total testesterone (TT) also showed a statistically significant group effect (one-way ANOVA, F(3, 20) = 131.787, *p* < 0.001). The observed effect size was large (η^2^ = 0.952), and the 95% confidence interval (0.882–0.966) confirmed the strength and consistency of the group differences. As depicted in Figure 4, no significant difference was observed between the FSH, LH, and testosterone levels in the blood of animals administered *Centella asiatica* and the healthy group (*p* > 0.05). FSH, LH, and testosterone levels in the blood of animals administered doxorubicin alone were significantly lower (*p* < 0.001) than those in the HC (Table 1). Furthermore, *Centella asiatica* significantly prevented the decrease in FSH, LH, and testosterone by DOX (*p* < 0.001).

### 2.5. Histopathological Results

**Figure 5:**
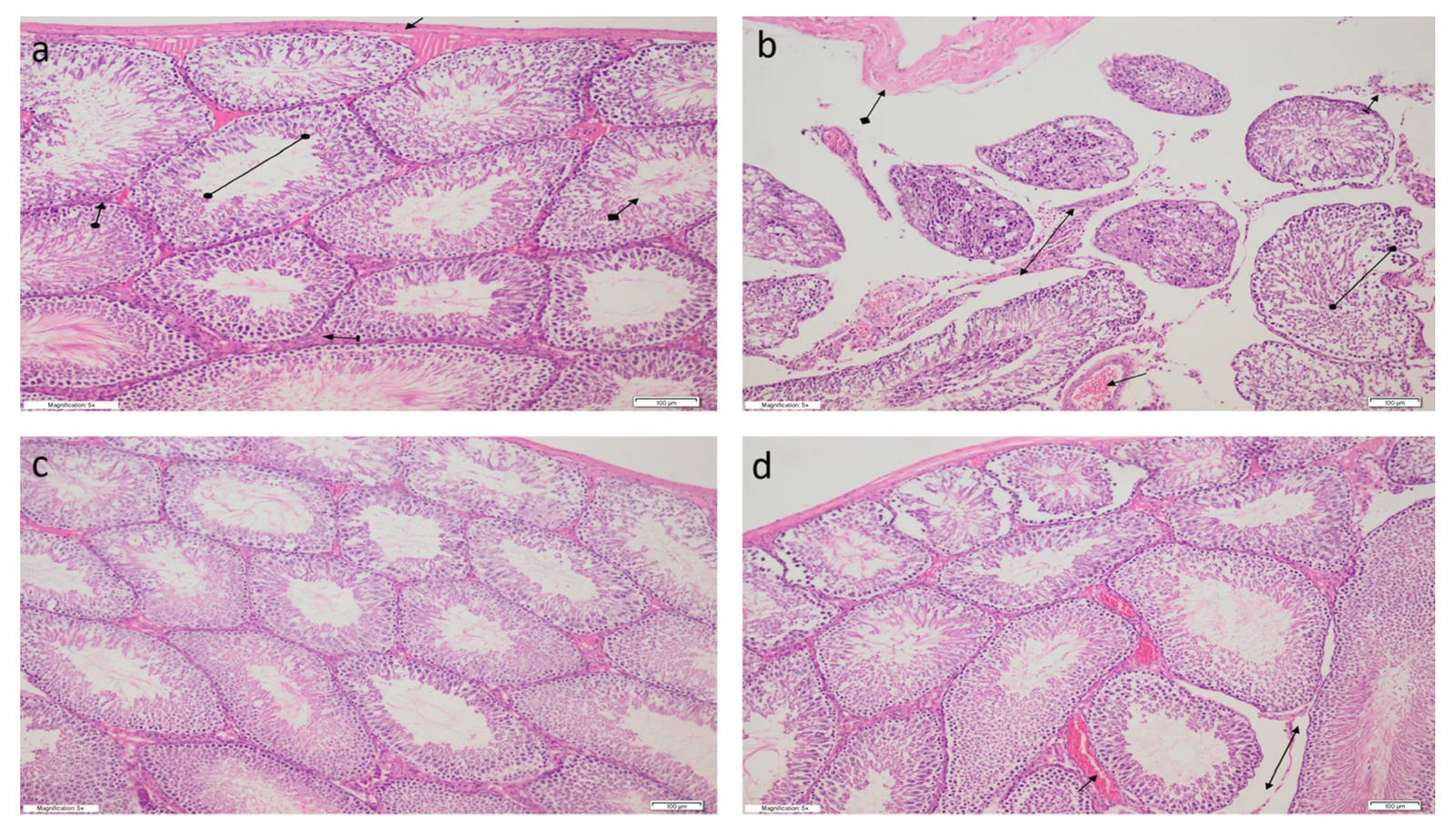
Hematoxylin–eosin-stained sections of rat testicular tissues (x100) (**a**–**d**). (**a**). Representative histopathological image of the HC group showing the tunica albuginea (→), interstitial connective tissue (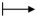), Leydig cells (●─►), seminiferous tubules (●─●), and spermatids (■─►). (**b**). DOX group showing tunica albuginea damage (■─►), seminiferous tubule destruction (●─●), interstitial connective tissue damage, hemorrhage and edema (↔), PMNL infiltration (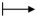), and dilated and congested blood vessels (→). (**c**). CA group showing a near-normal histological appearance. (**d**). CADX group showing dilated and congested blood vessels (→) and mild persistent interstitial connective tissue damage and edema (↔). Healthy Control (HC), *Centella asiatica* (CA), Doxorubicin (DOX), *Centella asiatica* + Doxorubicin (CADX), scale bar = 100 µm.

As illustrated in Figure 5a, the testicular tissue of the HC group showed a normal histological architecture, including the tunica albuginea, spermatid, seminiferous tubules, Leydig cells, and interstitial connective tissue. However, severe degenerative changes graded as Grade 3 including tunica albuginea damage, seminiferous tubule destruction, edema, vascular dilation and congestion, connective tissue loss, hemorrhage, and PMNL infiltration were evident in the DOX-treated group (Figure 5b). No histopathological alterations were observed in the group treated with *Centella asiatica* alone, which was scored as Grade 0 (Figure 5c). Conversely, *Centella asiatica* treatment effectively attenuated DOX-induced morphological damage, reducing the histopathological score to Grade 1, with only focal interstitial connective tissue damage, edema, and dilated/congested blood vessels persisting (Figure 5d; Table 2).

## 3. Discussion

In the present study, the potential protective efficacy of *Centella asiatica* against DOX-induced testicular injury in rats was evaluated through comprehensive biochemical and histopathological analyses. Our findings demonstrate that *Centella asiatica* exerts a robust protective effect against DOX-induced testicular damage. Although DOX is a potent anthracycline antibiotic widely utilized in cancer chemotherapy, its clinical application is critically limited by its severe toxicity toward testicular tissue [9]. Antineoplastic therapies are documented to negatively impact male reproductive capacity and spermatogenesis. Mechanistically, increased oxidative stress in germ cells have been identified as key pathways mediating this chemotherapy-induced gonadal toxicity [24]. Oxidative stress arises from an imbalance between pro-oxidant molecules and the antioxidant capacity of cells [25]. In this investigation, measured MDA levels were utilized as a dependable indicator to evaluate the degree of cellular and tissue injury induced by oxidative stress [26]. Our experimental results are consistent with the existing literature reporting that DOX administration adversely impacts testicular reproductive capacity through oxidative stress pathways, leading to impaired sperm parameters and histopathological cellular degradation [4]. In the DOX-treated group, the MDA level in the testicular tissue increased significantly compared to that in the control group. Furthermore, NO levels were measured to evaluate oxidative stress induced by DOX in the testes. Our findings revealed that NO levels were significantly elevated in the DOX group compared to those to in control group. As is well known, NO is a critical signaling molecule involved in various physiological and pathophysiological processes. Depending on the severity and duration of testicular ischemia, NO may mediate cell death through apoptosis or necrosis [27]. In an animal study, the DOX-treated group exhibited elevated NO levels and toxic tissue alterations compared with the control group [28].

Given that oxidative tissue damage is characterized by a reduction in antioxidant levels, testicular SOD activity was assessed to gauge the extent of DOX-related toxicity. The findings revealed a pronounced reduction in SOD levels within the testicular tissues of the DOX group, indicating a compromised antioxidant defense system. Furthermore, DOX-induced oxidative stress has been suggested to result from the suppression of cellular antioxidant defense mechanisms, including SOD and CAT. [10]. The literature further states that DOX toxicity increases oxidative stress, lipid peroxidation (LPO), and reactive oxygen and nitrogen species while reducing antioxidant defense systems [29]. In our study, TNF-α, IL-6, and IL-1β levels were measured in the testicular tissues of animals administered DOX to assess the inflammatory response. These parameters represent key pro-inflammatory cytokines secreted by macrophages and monocytes in response to various stimuli [30]. Previous studies have indicated that following DOX administration significant decreases in testosterone, GSH levels, and the activities of CAT, glutathione peroxidase (GPx), and SOD in rats were accompanied by significantly increased nitric oxide, MDA, FSH, and LH levels [31]. Our findings are consistent with the literature, showing a marked increase in TNF-α, IL-6, and IL-1β levels in the DOX group compared with the control group.

Previous studies have reported that DOX treatment reduces circulating TT levels, the primary male sex hormone [10]. In parallel with these data, our findings showed that TT levels were significantly decreased in the DOX-treated group compared to the control group. Thus, the notion that DOX may impair Leydig cell function, thereby reducing testosterone synthesis and negatively affecting male fertility, is supported [32]. Our results further showed that DOX significantly decreased FSH and LH levels. The reduction in LH levels despite low testosterone concentrations in the DOX group suggests a disruption of the negative feedback mechanism, given that testosterone production is regulated by LH secretion. Our findings are consistent with a previous study reporting that DOX treatment decreased serum FSH and LH levels along with total testosterone (TT) concentrations in male rats [33]. Although decreased testosterone would normally be expected to increase LH and FSH secretion through negative feedback, the concomitant reduction in all three hormones observed in the present study suggests that DOX may impair the regulation of the hypothalamic–pituitary–gonadal axis in addition to causing direct testicular injury. DOX-induced oxidative stress and inflammation have been reported to disrupt hypothalamic and pituitary function, potentially suppressing gonadotropin secretion [10]. Therefore, the simultaneous decline in testosterone, LH, and FSH may reflect combined central endocrine dysfunction and impaired testicular steroidogenesis rather than a typical compensatory response. This finding contrasts with previous research indicating hormonal imbalance and infertility [5], as LH and FSH levels did not increase despite decreased TT levels in our study [34].

CA, whose protective effects against DOX-related oxidative and inflammatory testicular injury were investigated in our study, has been reported to possess anti-inflammatory and antioxidant properties [35,36]. Our findings showed that, combined *Centella asiatica* and DOX administration inhibited the increase in MDA levels and the reduction in SOD levels. These data show that *Centella asiatica* preserves redox balance in testicular tissue and prevents its disruption by DOX. Furthermore, our results align with those of previous studies [37]. Treatment with *Centella asiatica* reduced NO levels in microglial cells exposed to oxidative stress and neuroinflammatory conditions, suggesting its potential to alleviate neuroinflammation. [38]. Our findings support the literature, as *Centella asiatica* prevented the DOX-induced increase in NO levels. As noted above, DOX impairs both redox homeostasis and the regulation of reproductive hormones [31]. Studies in the literature also indicate that *Centella asiatica* suppresses increases in pro-inflammatory cytokines [37,39,40]. Consistent with these findings, our data show that *Centella asiatica* extract significantly reduced the DOX-induced elevation of TNF-α, IL-1β, and IL-6 in testicular tissue.

*Centella asiatica* may exert its protective effects against DOX-induced testicular toxicity by reducing the generation of ROS and attenuating oxidative stress. The pathophysiology of DOX-induced testicular injury involves multiple mechanisms, including inflammatory activation, excessive production of ROS, autophagy dysregulation, and oxidative stress (4,5). The relationship between antioxidant supplementation and anticancer therapy remains controversial. While some hypotheses suggest that antioxidants may attenuate the efficacy of anticancer drugs by reducing oxidative stress-mediated cytotoxicity [41], other studies have demonstrated potential benefits, including improved survival, enhanced tumor responses, and reduced chemotherapy-related toxicity [42]. Therefore, tumor-bearing models are required to determine whether *CA* can protect normal tissues against DOX toxicity while preserving its antineoplastic efficacy.

In this study, we also evaluated the effects of *Centella asiatica* on spermatogenesis and hormones associated with the hypothalamic–pituitary–gonadal axis. Previous research has shown that circulating TT levels decrease with DOX treatment [10]. However, the literature suggests that *Centella asiatica* may impair spermatogenesis by affecting Leydig cells, exert adverse effects on Sertoli cells, and cause antifertility effects associated with triterpenoids such as asiaticoside [43,44]. In contrast to prior studies [45], our findings revealed that FSH, LH, and TT levels, which decreased following DOX administration, approached normal levels with *Centella asiatica* treatment. The discrepancy between our findings and the previous report by Heidari et al. may be related to differences in the experimental design, particularly the duration of *Centella asiatica* administration. Heidari et al. administered different doses of *Centella asiatica* for 60 days, and the prolonged exposure may have contributed to alterations in spermatogenic processes. In contrast, our study used *Centella asiatica* administration for a shorter period (7 days) in a DOX-induced testicular injury model. Under these conditions, the antioxidant and anti-inflammatory properties of *Centella asiatica* may have counteracted DOX-induced oxidative damage and preserved testicular tissue integrity. Therefore, the different outcomes may be attributed to variations in dose, duration of exposure, experimental model, and biological context.

Histopathologically, pronounced destruction of the seminiferous tubules, edema, dilated congested blood vessels, connective tissue loss, hemorrhage, and PMNL infiltration were observed in the testicular tissues of the DOX group [2]. The literature further indicates that DOX causes atrophy and degeneration in seminiferous tubules as well as loss of spermatogenic cells [46]. In contrast, the testicular tissues of animals treated with *Centella asiatica* alone displayed a morphology similar to that of the healthy group. The fact that *Centella asiatica* mitigated DOX-induced the morphological changes in the testicular tissue does not align with previous literature [45]. Although the CADX group demonstrated near-complete histological recovery, several biochemical parameters remained significantly different from the control groups. This finding is not unexpected, as histopathological evaluation reflects structural recovery, whereas biochemical markers are more sensitive to persistent oxidative stress, inflammatory activity, and cellular dysfunction. Therefore, morphological improvement may precede complete biochemical normalization. Despite differences in the degree of recovery, both histopathological and biochemical findings consistently demonstrated the protective effects of *Centella asiatica*.

This study has several limitations. First, as an experimental animal study, the findings cannot be directly generalized to humans; therefore, clinical studies are required to confirm their translational relevance. Second, only specific doses and durations of DOX and *Centella asiatica* administration were evaluated, and the effects of different doses, treatment durations, and administration routes remain to be investigated. Furthermore, *Centella asiatica* was administered before and during DOX exposure; thus, only its preventive effects were assessed, while its therapeutic potential after established testicular injury was not examined. Since antioxidant interventions may be initiated after chemotherapy in clinical practice, future studies should evaluate post-treatment protocols to determine whether *Centella asiatica* can reverse or alleviate pre-existing testicular damage.

Another limitation is the lack of an a priori power analysis. The sample size was determined based on previous experimental studies with similar designs and in accordance with the principles of animal reduction within the framework of the 3 R principles (Replacement, Reduction, and Refinement). Nevertheless, future studies incorporating formal sample size calculations and larger cohorts are warranted to further validate and strengthen these findings. In addition, the absence of a positive control group limits direct comparisons between the protective efficacy of *Centella asiatica* and established protective agents. Future studies including validated reference compounds are needed to better define the relative protective potential of *Centella asiatica*. Another limitation of this study is direct fertility-related outcomes, including sperm parameters, reproductive organ weights, and mating outcomes, were not evaluated. Therefore, the findings are limited to the biochemical, endocrine, and histopathological protective effects of *Centella asiatica*. However, the underlying molecular mechanisms responsible for the protective effects of *Centella asiatica* were not investigated in the present study. Further studies evaluating specific signaling pathways, such as Nrf2/HO-1, NF-κB, apoptotic markers, and steroidogenic-related proteins, are required to clarify the molecular basis of its protective effects. Finally, although the whole *Centella asiatica* extract was evaluated rather than individual bioactive compounds, the observed protective effects may result from the synergistic actions of multiple phytochemicals. However, the lack of detailed phytochemical standardization represents a limitation, and further investigations are required to determine the contribution of specific compounds, such as asiatic acid and asiaticoside, to the protective effects of *Centella asiatica*.

## 4. Materials and Methods

### 4.1. Experimental Animals

Within the scope of the study, a total of 24 adult male albino Wistar rats (258–265 g) obtained from the Erzincan Binali Yıldırım University Medical Experimental Application and Research Center were used. The animals were housed in a controlled environment with a stabilized temperature of 22 ± 2 °C and relative humidity of 30–70%, a 12-h light/12-h dark cycle, with ad libitum access to standard pellet feed and tap water. The study design and experimental practices were conducted in full compliance with the European Union Directive 2010/63/EU on the protection of animals used for scientific purposes, the ARRIVE reporting guidelines.

### 4.2. Drug and Agents

In this study, doxorubicin hydrochloride (50 mg/25 mL) was obtained from Saba Drug Industries (Adrimisin^®^, Istanbul, Türkiye), *Centella asiatica* extract was obtained from Herb Pharm (Williams, OR, USA), and thiopental sodium was obtained from IE Ulagay (Istanbul, Türkiye). The extract was prepared from certified organic dry leaves/aerial parts of *Centella asiatica* (L.) Urb. at a herb-to-menstruum ratio of 1:5. The menstruum consisted of certified organic cane alcohol (60–70%), distilled water, and vegetable glycerin. According to the manufacturer’s analytical specifications, the identity and quality of the extract were validated using High-Performance Thin-Layer Chromatography (HPTLC) fingerprinting against standard reference compounds (specifically for pentacyclic triterpenes including asiaticoside, madecassoside, asiatic acid, and madecassic acid).

### 4.3. Animal Groups

Rats were randomly assigned to four groups (N = 6): healthy control (HC), *Centella asiatica* group (CA), doxorubicin (DOX), and *Centella asiatica* combined with DOX (CADX). The sample size was determined based on previous experimental studies using similar rat models of doxorubicin-induced testicular injury [47].

### 4.4. Experimental Design

To comply with the 3R principles and ensure reliable experimental outcomes, the minimum number of animals required for the study was used [48]. Predefined exclusion criteria included abnormal clinical signs before the experiment and unexpected events during or after the procedures, such as mortality, severe distress, major complications, protocol deviations, incomplete treatment administration, excessive weight loss (>15–20%), or tissue damage affecting analyses. No animals met the exclusion criteria, and all were included in the final evaluation.

### 4.5. Experimental Procedure

*Centella asiatica* 200 mg/kg was administered orally via gavage [18] to the CA and CADX groups for seven consecutive days [47]. The pharmacological dose of *Centella asiatica* has previously been reported to exhibit significant antioxidant and anti-inflammatory effects in experimental studies [18,49]. The 7-day administration period of *Centella asiatica* was selected based on the acute nature of the DOX-induced testicular injury model and the aim of evaluating the early protective effects of *Centella asiatica* during DOX exposure. The HC and DOX groups received oral saline (0.9% Sodium chloride). One hour after *Centella asiatica* or saline administration, the DOX and CADX groups received intraperitoneal DOX (7.5 mg/kg) on days 1, 4, and 7, following the protocol described by Gurel et al. The DOX administration schedule used in our study was determined based on previous experimental studies. It has been demonstrated that doxorubicin administration on days 1, 4, and 7 is sufficient to induce testicular toxicity in rats by causing oxidative stress and histopathological changes in testicular tissue [50]. The doses of *Centella asiatica* (200 mg/kg) and DOX (7.5 mg/kg) were selected based on previous experimental studies reporting their effectiveness in antioxidant and toxicity models, respectively. The present study aimed to evaluate the protective efficacy of *Centella asiatica* against DOX-induced testicular injury; therefore, a single dose of each compound was investigated. Animals were observed daily for general health and signs of distress throughout the experimental period. No mortality or unexpected adverse events occurred, and no animals were excluded from the study. Body weight was not monitored. Following the experimental period, rats were euthanized with thiopental sodium 50 mg/kg, and the testicular tissues were rapidly excised for further analysis. This procedure was conducted in accordance with institutional animal ethics guidelines to minimize pain and distress. Pro-inflammatory cytokines (TNF-α, IL-1β, IL-6) and oxidative stress parameters (MDA, SOD, NO) were assessed in the harvested testicular samples to determine the extent of tissue damage. The serum concentrations of total TT, LH, and FSH were assessed in pre-euthanasia blood samples. Simultaneously, the tissues were subjected to microscopic evaluation via histopathological analysis. Biochemical and hormonal analyses were conducted under single-blind conditions, with researchers responsible for the measurements unaware of the experimental group assignments.

### 4.6. Biochemical Analyses

#### 4.6.1. Measurement of MDA and SOD Determination in the Testicular Tissue

The testicular tissues were washed, frozen in liquid nitrogen, powdered, and homogenized. Supernatants were used for malondialdehyde (MDA, μmol/mg protein; Cat No: 10009055; Sensitivity: 0.625 µM; Detection Range: 0.625–50 µM; Intra/Inter Assay CV: <8%/<5%) and Superoxide Dismutase (SOD, U/mg Protein; Cat No: 706002; Sensitivity: 0.005 U/mL; Detection Range: 0.005 U/mL; Intra/Inter Assay CV: <3.2%/<3.7%) measurements using commercial enzyme-linked immunosorbent assay (ELISA) kits (Cayman Chemical, Ann Arbor, MI, USA). Total protein levels were quantified spectrophotometrically at 595 nm using the method described by Marion M. Bradford [51].

#### 4.6.2. Measurement of NO Determination in the Testicular Tissue

Nitric oxide (NO, μmol /mg protein) levels were determined using the Griess method [52]. In this two-stage method, nitrate was first reduced to nitrite using the enzyme nitrate reductase. In the second stage, nitrite was converted to a purple azo compound by adding Griess reagent. The absorbance of the compound at 540 nm was measured photometrically, and the nitrite concentration was determined.

#### 4.6.3. Measurement of TNF-α, IL-1β, and IL-6 Determination in the Testicular Tissue

Tissue samples were first weighed, finely cut, rapidly frozen in liquid nitrogen, and homogenized using a mortar and pestle. After thawing, the samples were stored at 2–8 °C. Phosphate-buffered saline (PBS, pH 7.4) was added at a 1:10 (*w*/*v*) ratio and mixed by vortexing for 10 s. The homogenates were centrifuged at 10,000× *g* for 20 min. Tissue TNF-α, IL-1β, and IL-6 levels were determined using commercial ELISA kits (Hangzhou Eastbiopharm Co., Ltd., Hangzhou, China) according to the manufacturer’s protocols. The levels of Tumor Necrosis Factor α (TNF-α, ng/tissue; Cat no: CK-E30636; Sensitivity: 2 pg/mL; Detection Range: 5–1000 pg/mL; Intra/Inter Assay CV: <8%/<10%), interleukin 1β (IL-1beta, pg/tissue Cat No: CK-E30635; Sensitivity: 1 pg/mL; Detection Range: 2.5–500 pg/mL; Intra/Inter Assay CV: <8%/<10%) and interleukin 6 (IL-6, ng/tissue, Cat No: CK-E30640; Sensitivity: 2 pg/mL; Detection Range: 5–1000 pg/mL; Intra/Inter Assay CV: <8%/<10%) were measured.

#### 4.6.4. Serum Hormone Analysis

ELISA kits (Hangzhou Eastbiopharm Co., Ltd., Hangzhou, China) were employed to detect follicle-stimulating hormone (FSH, ng/mL; Cat No:CK-E30597; Sensitivity: 0.12 mIU/L; Detection Range: 0.2–60 mIU/L; Intra/Inter Assay CV: <8%/<10%), luteinizing hormone (LH, mlU/mL; Cat No: CK-E90904; Sensitivity: 0.11 mIU/L; Detection Range: 0.2–60 mIU/L; Intra/Inter Assay CV: <8%/<10%), and testosterone (ng / mL; Cat No: E90243; Sensitivity: 0.25 nmol/L; Detection Range: 0.5–100 nmol/L; Intra/Inter Assay CV: <8%/<10%).

#### 4.6.5. Histopathological Analysis

Following fixation in a 10% formalin solution, the tissues were washed overnight. Dehydration was subsequently performed using a graded alcohol sequence, followed by transparency with xylene and final embedding in paraffin to prepare them for sectioning. After H&E staining of the 4–5 µm paraffin sections, histopathological evaluation was performed by a blinded expert. For each animal, one microscopic field from the central region and five microscopic fields from the peripheral regions of the testicular tissue section were evaluated. The tissue sections were coded before examination, and the evaluator was unaware of the experimental groups during microscopic assessment and scoring. Semi-quantitative scoring (0–3) was used to evaluate destruction, connective tissue changes, and vascular dilatation. Histopathological changes were graded as follows: Grade 0, absent (no histopathological findings); Grade 1, mild (findings present in <25% of the examined fields); Grade 2, moderate (findings present in 25–50% of the fields); and Grade 3, severe (findings present in >50% of the fields) [53]. All imaging and documentation were conducted using the Olympus DP2-SAL (Ver. 3.3.1.198, Evident-Olympus, Tokyo, Japan) software.

### 4.7. Statistical Analysis

All statistical analyses were performed using IBM SPSS Statistics version 22 (IBM, Armonk, NY, USA), and graphical presentations were generated using GraphPad Prism 9 (GraphPad Software, San Diego, CA, USA). The distribution of biochemical variables was assessed using the Shapiro–Wilk test, and homogeneity of variances was evaluated using Levene’s test. For normally distributed data, intergroup comparisons were conducted using one-way analysis of variance (ANOVA), followed by Tukey’s honestly significant difference (HSD) test for post hoc analysis. Biochemical data are presented as mean ± standard deviation (SD). Histopathological semi-quantitative scores were analyzed using the Kruskal–Wallis test followed by Dunn’s multiple comparisons test. Histopathological scores are presented as median (Q1–Q3). Statistical significance was accepted at *p* < 0.05.

## 5. Conclusions

This study aimed to evaluate the potential protective effects of the antioxidant agent *Centella asiatica* against DOX-induced testicular toxicity. Prolonged DOX exposure compromised testicular tissue integrity by elevating oxidant markers and pro-inflammatory cytokines, depleting antioxidant defenses, and inducing reproductive hormone dysfunction. Furthermore, DOX administration severely disrupted normal testicular histomorphology. Conversely, CA treatment effectively mitigated the severity of DOX-induced oxidative, inflammatory, and morphological damage within the testicular tissue, while significantly ameliorating reproductive hormone imbalances. Collectively, our findings suggest that *Centella asiatica* holds substantial therapeutic potential as a protective agent against DOX-induced male reproductive organ injury and subsequent dysfunction.

## Figures and Tables

**Table 1:** Analysis results of biochemical parameters in testicular tissue and serum.

Groups	Biochemical Parameters Mean ± Standard Deviation								
	Testicular Tissue						Serum		
	MDA (μmol/mg Protein)	SOD (U/mg Protein)	NO (μmol/mg Protein)	TNF-α (ng/Tissue)	IL-1β (pg/Tissue)	IL-6 (ng/Tissue)	FSH (ng/mL)	LH (mlU/mL)	Testosterone (ng/mL)
HC	2.36 ± 0.35	3.49 ± 0.33	0.57 ± 0.10	43.20 ± 3.30	24.13 ± 3.17	10.54 ± 0.90	0.79 ± 0.02	40.56 ± 3.67	7.11 ± 0.51
CA	2.32 ± 0.26	3.48 ± 0.48	0.58 ± 0.13	40.26 ± 3.84	23.44 ± 4.43	9.64 ± 2.21	0.77 ± 0.02	41.35 ± 3.09	7.12 ± 0.62
DOX	4.49 ± 0.32 *^,≠^	1.52 ± 0.27 *^,≠^	1.42 ± 0.15 *^,≠^	122.05 ± 5.35 *^,≠^	70.29 ± 3.94 *^,≠^	20.81 ± 2.61 *^,≠^	0.23 ± 0.02 *^,≠^	21.37 ± 2.87 *^,≠^	2.39 ± 0.32 *^,≠^
CADX	3.30 ± 0.38 *^,≠,ε^	2.60 ± 0.08 *^,≠,ε^	0.86 ± 0.08 *^,≠,ε^	88.64 ± 3.35 *^,≠,ε^	41.39 ± 5.44 *^,≠,ε^	16.43 ± 0.82 *^,≠,ε^	0.55 ± 0.03 *^,≠,ε^	32.68 ± 3.09 *^,≠,ε^	4.52 ± 0.46 *^,≠,ε^

Graph Abbreviations: HC: Healthy Control, CA: *Centella asiatica* extract, DOX: Doxorubicin, CADX: *Centella asiatica* extract + Doxorubicin, MDA: Malondialdehyde, SOD: Superoxide dismutase, NO: Nitric oxide, TNF-ɑ: Tumor necrosis factor-alpha, IL-1β: Interleukin-1 beta, IL-6: Interleukin-6, FSH: Follicle-stimulating hormone, LH: Luteinizing hormone. *p* < 0.001 * vs. HC (CA, DOX, CADX); ^≠^ vs. CA extract (DOX, CADX), ^ε^ CADX vs. DOX.

**Table 2:** Histopathological analysis in rat testicular tissues.

	Median (Quartile 1–Quartile 3)					
Groups	Destruction	Oedema	Dilatation/Congestion Blood Vessels	Connective Tissue Changes	Hemorrhage	PMNL Infiltration
HC	0 (0–0)	0 (0–0)	0 (0–0)	0 (0–0)	0 (0–0)	0 (0–0)
CA	0 (0–0)	0 (0–0)	0 (0–0)	0 (0–0)	0 (0–0)	0 (0–0)
DOX	3 (2–3) *^,‡^	3 (3–3) *^,‡^	3 (2–3) *^,‡^	3 (2–3) *^,‡^	3 (2–3) *^,‡^	3 (2–3) *^,‡^
CADX	0 (0–1) ^‡,γ^	1 (0–1) *^,‡,γ^	1 (0–2) *^,‡,γ^	1 (0–1) *^,‡,γ^	0 (0–1) ^γ^	0 (0–1) ^γ^

Graph differences were analyzed by Kruskal–Wallis followed by Dunn’s test. Histopathological grading: 0, none; 1, mild; 2, moderate; and 3, severe damage. * *p* < 0.05 vs. HC, ^‡^ *p* < 0.05 vs. CA, ^γ^ *p* < 0.05 DOX. Abbreviations: HC: Healthy Control, CA: *Centella asiatica*, DOX: Doxorubicin, CADX: *Centella asiatica* + Doxorubicin, PMNL: Polymorphonuclear leukocyte.

## Data Availability

The data supporting the findings of this study are available from the corresponding author upon reasonable request, subject to ethical considerations. This article is a revised and expanded version of the abstract titled ‘Investigation of the Protective Effect of *Centella asiatica* against Doxorubicin-Induced Testicular Damage in Rats’, which was presented at the 7th International Congress on Innovative Approaches in Medical and Health Sciences in Istanbul/Türkiye (4–5 December 2025).
